# The prevalence of workaholism: a systematic review and meta-analysis

**DOI:** 10.3389/fpsyg.2023.1252373

**Published:** 2023-10-30

**Authors:** Filip Borgen Andersen, Merjem Emma Torlo Djugum, Victoria Steen Sjåstad, Ståle Pallesen

**Affiliations:** Department of Psychosocial Science, University of Bergen, Bergen, Norway

**Keywords:** work addiction, workaholism, prevalence, frequency, epidemiology, systematic review, meta-analysis

## Abstract

The present study represents the first meta-analysis and systematic review on the prevalence of workaholism. It also investigated if sample size, representativeness, and instrument moderated the prevalence estimates. The analysis was pre-registered at PROSPERO (CRD42023395794). We searched Web of Science, PubMed, CINAHL, Embase, PsychInfo. BASE, MedNar, NYAM, OPENGREY, OpenMD and included the first 200 searches on Google scholar as gray literature [search string: “(workaholi* OR “work addict*”) AND (prevalence* OR incident* OR frequen* OR cut-off OR epidem*)]. The search yielded 42 studies to be included, in addition to 11 studies identified using other methods. Two independent raters went through the searches, extracted information and evaluated risk of bias, resulting in agreement ratings of 92.4%, 84.9%, and 87.0%, respectively. The inclusion criteria were studies reporting original data on the prevalence of workaholism written in any European language. Criteria which led to exclusion were conference abstracts, usage of secondary data, purposive sampling of workaholics, qualitative research and pre-determined cut-off based on distribution. Risk of bias of the included articles was evaluated through a checklist. Most of the included studies had a moderate risk of bias. Of the 663 records identified, a total of 53 studies were included, 10 of these being nationally representative with all studies in total amounting to 71,625 participants from 23 countries. The pooled workaholism prevalence was 15.2% (95% CI = 12.4–18.5), which was adjusted to 14.1% (95% CI = 11.2–17.6) following a trim-and-fill adjustment for publication bias. The meta-regression revealed that studies with representative samples reported lower prevalences than those based on non-representative samples, and that studies based on the Dutch Work Addiction Scale yielded higher prevalences than studies employing the Bergen Work Addiction Scale. The regression model explained 29% of the variance implying that a vast amount was still unexplained, and that future research would benefit from the inclusion of other moderators.

## Introduction

For most people, work plays a prominent role in terms of fulfilling social and psychological needs (Eliason and Storrie, 2009; Clark et al., 2016). Work imbues life with meaning, generates monetary value, establishes structure, fosters social connections, forges status, and promotes activity (Jahoda, 1981). The majority of workers therefore tend to experience positive outcomes from working, however a small minority seems to engage in excessive and uncontrollable work behavior. Such individuals are often driven by internal and external factors that make them compelled to work and are referred to as workaholics (Andreassen, 2013). The term workaholism was historically introduced by Oates (1971) who defined it as “addiction to work, the compulsive and uncontrollable need to work incessantly.” More recent definitions of workaholism have expanded upon Oates’ dimensions to provide a more nuanced understanding of the concept. One such comprehensive and relatively agreed upon definition implies that workaholism represents a compulsive and extreme need to work, characterized by an unrelenting drive to work hours on end, take on more work responsibility and prioritize work over different areas of life, such as family and friends (Andreassen, 2013). Workaholism can have negative psychological, physical and social effects on afflicted individuals and those close to them and has also been linked to detrimental organizational outcomes. Most studies on workaholism focus on mechanisms and outcome variables (Hu, 2018), hence data on the number of people affected by workaholism is scarce which this topic share with other work-and organizational problems (Luckhaupt and Calvert, 2010).

The latter creates a gap that reduces the likelihood of governmental and stakeholder recognition which in turn restricts implementation of intervention strategies and practical guidelines that aim to combat workaholism. In order to reduce this gap, the goal of the present study was to pool estimates of workaholism to produce a single estimate reflecting the significance and magnitude of workaholism. This can be difficult due to three reasons: (i) there is no clear definitional consensus between experts regarding the construct, (ii) there is no established consensus in terms of how to assess workaholism and the appropriate cut-off differentiating between regular and pathological work-related behavior, and (iii) the majority of studies on workaholism are not based on representative samples. Consequently, this limits the generalizability of findings and hampers comparisons across studies. A meta-analytic synthesis can circumvent limitations associated with single studies and may identify additional factors that act as moderators of workaholism prevalence estimates. Against this backdrop the second goal of the present study was to investigate if three *a priori* decided moderators (sample size, representativeness, and instrument) moderate the prevalence estimates. With this approach the goal of the present article was to add to the existing theoretical foundation of workaholism research and as such contribute to the future development of relevant instruments and preventive measures.

### Workaholism conceptualization

In recent times, workaholism has become a part of most people’s vocabulary and during the past decades research into the subject matter has spurred (Andreassen, 2013). With this newfound interest the conceptualization of workaholism has undergone drastic changes since its initial presentation in literature. Early research on workaholism often classified workers as workaholics if they partook in work-activities over a certain number of hours, e.g., 50 h/week (Mosier, 1983). However, several non-workaholic workers would fit into this classification due to factors such as cultural practices and occupational norms (Kang, 2020). Researchers have since developed more nuanced definitions that take these factors into account and incorporate additional features of workaholism (e.g., compulsion, drive) when assessing the construct. Subsequently, most research afterwards leaned into an understanding of workaholism that emphasized a multidimensional approach (Andreassen, 2013). Spence and Robbins (1992) suggested that workaholism consists of three dimensions; work involvement, drive to work and work enjoyment. Individuals who scored high on all dimensions were classified as enthusiastic work addicts, whereas those with a high score on the two first and a low score on the last dimension were classified as non-enthusiastic work addicts (Spence and Robbins, 1992). By time the multidimensional perspective was discarded as the emphasis on work enjoyment was deemed irrelevant (Mudrack, 2006) and the work involvement subscale was found to be invalid (Andreassen et al., 2013b). More recent conceptualizations asses workaholism with dimensions that correlate substantially (Schaufeli et al., 2009) and unidimensional workaholism instruments have been developed (Andreassen et al., 2012). The terms “enthusiastic work addiction” or “positive workaholism” seem now to have been replaced by the term “work engagement” (Taris et al., 2020). Hence, workaholism now seems reserved to the previously used term “non-enthusiastic work addiction” (Andreassen, 2013). Still, some argue that the term “workaholism” highlights the positive elements of work (e.g., benefits), whereas “work addiction” mostly reflects the negative elements of work (Griffiths et al., 2018). Other scholars maintain that one should not differentiate between the two and that both “workaholism” and “work addiction” refers to the same construct (Andreassen et al., 2018b). Some argue that workaholism is best described as an addiction (Andreassen et al., 2012) while others believe it is better understood as a spectrum of work-behavior (e.g., Spence and Robbins, 1992). In summary, the current state-of-science regarding the conceptualization of workaholism is somewhat fragmented by lack of common ground among experts. The differences in conceptualization have accumulated into a selection of diverse workaholism measurements.

### Workaholism measurements

Researchers and clinicians have since the 1980s developed different workaholism screening measurements (Andreassen, 2013). Here we present a short overview of the most commonly used. The Work Addiction Risk Test (WART) is a self-report questionnaire that consists of 25 items, each rated on a four-point Likert scale, and measures five dimensions of workaholism: *Control* (seven items: e.g., “I get impatient when I have to wait for someone else or when something takes too long”), *compulsive tendencies* (nine items: e.g., “I feel guilty when I am not working on something”), *inability to delegate* (one item: e.g., “I prefer to do most things myself rather than ask for help”), *impaired communication/self-absorption* (five items: e.g., “I forget, ignore, or minimize birthdays, reunions, anniversaries, or holidays”) and *self-worth* (two items: e.g., “I am more interested in the final results of my work than in the process”; Flowers and Robinson, 2002). These dimensions were based on the developer’s definition of workaholism as “*the overindulgence in and preoccupation with work, often to the exclusion and detriment of the workaholic’s health, intimate relationships, and participation in child rearing”* (Flowers and Robinson, 2002). Individuals who have an overall score of 67–100 on the WART are typically classified as having high workaholic tendencies. The WART has been used in several studies and has demonstrated good internal consistency (Robinson, 1999). However, the measurement has faced criticism for its factor structure as more recent literature has found that the WART fails to capture the core aspects of workaholism (Schaufeli et al., 2009).

Another measurement that was used quite frequently, albeit less in recent times, is the Workaholism Battery, also known as WorkBAT. It contains 75 items scored on a five-point Likert scale and conceptualizes workaholism into three dimensions consisting of *drive* (seven items: e.g., “I seem to have an inner compulsion to work hard”), *work involvement* (eight items: e.g., “Wasting time is as bad as wasting money”) and *work-enjoyment* (10 items: e.g., “I like my work more than most people do”). Work involvement is described as one’s psychological involvement with work, work enjoyment reflects the extent of emotional satisfaction one gains from working, and drive refers to the internal motivation to work (Aziz et al., 2013). Individuals who score above the mean on drive and work-involvement while scoring below the mean on work-enjoyment are classified as non-enthusiastic workaholics (Spence and Robbins, 1992). These non-enthusiastic workaholics are what we in common terms today would consider work addicts.

A more recently developed measurement, the Dutch Work Addiction Scale (DUWAS) consists of 10 items that measure two aspects of workaholism, *working compulsively* (five items: e.g., “I feel guilty when I take time off work”) *and working excessively* (five items: e.g., “I seem to be in a hurry and racing against the clock”). Each item is scored on a four-point Likert scale ranging from (*almost*) *never* (1) to *(almost) always* (4). The two dimensions rely on Taris and Schaufeli’s conceptualization of workaholism as an overwhelming inner drive to work extremely hard comprised of *working compulsively* and *working excessively* (Schaufeli et al., 2008a). The latter captures the behavioral aspect of workaholism and *working compulsively* encapsulates the cognitive aspect of workaholism (Schaufeli et al., 2009). People scoring above the 75th percentile on both scales are generally classified as workaholics. The DUWAS was investigated in Dutch and Japanese workers where the scale showed high internal consistency. Some experts have critiqued the aforementioned measures for their lack of theoretical anchoring with the addiction field (Andreassen et al., 2012).

In an effort to overcome this limitation, Andreassen et al. (2012) constructed the Bergen Work Addiction Scale (BWAS), a self-report measure that aligns with addiction theory and comprises seven items, one reflecting each of the seven addiction components outlined by previous research (Griffiiths, 1996, 2005), including *mood modification* defined as the activity’s ability to moderate or improve mood (“Work in order to reduce feelings of guilt, anxiety, helplessness and depression”)*, tolerance* defined as the need for increasing amounts of the given activity to achieve the initial effect (“spend much more time working than initially intended”)*, salience* defined as the activity’s command of behavior and thinking (“think of how you can free up more time to work”)*, conflict* defined as the activity’s ability to cause conflicts in social relationships and other activities (“deprioritize hobbies, leisure activities, and exercise because of your work”)*, withdrawal* conceptualized as the incidence of unpleasant feelings when the activity is discontinued or abruptly reduced (“become stressed if you are prohibited from working”)*, problems* defined as health problems or other difficulties linked to the addiction (“work so much that it has negatively influenced your health”), *and relapse* defined as a tendency for reversion to previous patterns of the activity after abstinence (“have been told by others to cut down on work without listening to them”; Andreassen et al., 2012). The response alternatives range from never (1) to always (5). Individuals who respond “often” or always” on 4 out of 7 items are considered to be work addicts. BWAS has been found to have good psychometric properties (Andreassen et al., 2012; Bellali et al., 2022).

Summarized, the current measurements focus on different aspects of workaholism. Some primarily view workaholism as an obsession or compulsion (e.g., DUWAS) while others highlight workaholism as an addiction (e.g., BWAS). While most of the measurements evaluate workaholism as a multidimensional concept (e.g., WART, WorkBAT, DUWAS), a minority assess it along a single dimension (e.g., BWAS; Andreassen, 2013). Despite the fact that researchers have created multiple measurements for assessing workaholism, a majority of these lack clear theoretical anchoring, are devoid of robust theoretical foundations, and demonstrate inadequate convergent validity among themselves (Andreassen et al., 2013b). It is important for workaholism measurements to be valid and reliable if they are to precisely identify workaholics, assess workaholism prevalences and link workaholism to various antecedents and consequences. By analyzing whether the measurements moderate the prevalence of workaholism one can investigate informal and formal assumptions about the validity and usefulness of the instruments.

### Correlates of workaholism

A large number of studies on workaholism demonstrate consistent relationships with impaired physical and mental health (Akutsu et al., 2022). Working like a workaholic have for example been associated with anxiety (Matsudaira et al., 2013), burnout (Galdino et al., 2021), cardiovascular disease (Balducci et al., 2021), depression (Dutheil et al., 2020 Yang et al., 2020), and sleeping problems (Andreassen et al., 2007 Salanova et al., 2016). Apart from depression and anxiety several studies attest to positive relationships with other types of psychiatric symptoms (e.g., Andreassen et al., 2016; Clark et al., 2016). Furthermore, workaholics are more likely to report lower-life satisfaction and overall well-being in comparison to average workers (Bonebright et al., 2000; Shimazu et al., 2015). As for psychosocial correlates, workaholism is positively associated with work–family conflicts (Andreassen et al., 2013a; Chang et al., 2022) and partners’ marital disaffection (Robinson et al., 2006). In terms of organizational outcomes many studies attest to positive associations between workaholism and negative organizational outcomes such as low job-satisfaction (Haar and Roche, 2013), job-stress (Sarfaraz et al., 2022) and work-related accidents (Andreassen et al., 2018a).

### Present meta-analytic research on workaholism

Prior meta-analytic studies on workaholism have mostly focused on its correlates. Some have assessed the link between workaholism and personality (e.g., Patel et al., 2012; Clark et al., 2016; Kun et al., 2020b). Others have chosen to direct attention toward workaholism and specific work-related constructs such as work performance (Cheng and Gu, 2022) and work engagement (Lee et al., 2022). To our knowledge there has not yet been conducted a meta-analysis on the prevalence of workaholism. It is reasonable to expect that estimates of workaholism vary in terms of methodological factors such as representativeness which may influence the estimates. A previous meta-analysis on the prevalence of compulsive buying did show higher rates in non-representative samples compared to representative samples (Maraz et al., 2016). It is therefore sensible to assume that representative studies on workaholism report lower rates of workaholism than studies based on non-representative samples. In the present article a study is considered representative of the population when findings are derived from a sample that can be applied to the broader target population—defined as the group of people whom the investigators seek to a make inference about (Rudolph et al., 2023). Studies based on representative samples, reflecting diverse populations and workplaces, are likely to produce prevalence estimates that are closer to the true prevalence in the broader populations. These studies are crucial for obtaining the most valid estimates (Richiardi et al., 2013). In a similar vein small-sample effect, e.g., higher effects and prevalences in small samples (Richter et al., 2019) may also influence the prevalence estimates. Although most likely less influential in prevalence studies, the present meta-analysis will still examine sample size as a potential moderator.

### The prevalence of workaholism

Studies on workaholism have found prevalences ranging from 1.5% (Demetrovics et al., 2022) to 44.9% (Azevedo and Mathias, 2017). The differences in reported prevalences are wide and in this subsection, we will present several studies that report varying rates of workaholism. In a study conducted in the US, it was estimated that around 10% of population could be suffering from workaholism (Sussman et al., 2011). In South-Korea some studies based on nationally representative samples of Korean workers report prevalence rates ranging from 6.7% (Park et al., 2020) to 39.7% (Kang, 2020). Since the two latter samples share major similarities, the disparity between the reported prevalence is somewhat unexpected. Still, one potential reason for the disparity may be authors’ utilization of different workaholism measures.

A recent study conducted in Egypt utilizing the DUWAS reported a workaholism prevalence of 33.0% in a sample of university staff (Allam et al., 2021). A similar study from Brazil found a workaholism prevalence of 35.5% (Galdino et al., 2021). The DUWAS was originally created and validated through two samples with one of them being Japanese workers (Schaufeli et al., 2009). Accordingly, most studies that assess workaholism in Japan employ the DUWAS. A study by Kubota et al. (2010) found a workaholism prevalence of 28.5% in a sample of Japanese nurses. In another study which employed the DUWAS to assess workaholism in a group of Japanese workers a prevalence rate of 29.4% was reported (Shimazu et al., 2011). Other studies employing the BWAS with nationally representative samples of Norwegian workers report prevalences stretching from 7.8% (Andreassen et al., 2016) to 8.3% (Andreassen et al., 2014). Hence, on the face of it, there seems to be a large disparity between reported frequency rates of workaholism.

Some measurements seem to regularly capture higher frequencies of workaholism compared to others. This difference may partially be explained by the conceptualization reflected by the instruments and the representativeness of the samples assessed (e.g., Kubota et al., 2010). Several studies have found inconsistent correlations between different operationalizations of workaholism (e.g., Andreassen et al., 2013b; Molino et al., 2022). Relatedly, studies have shown that DUWAS correlates positively with work engagement (WE) while BWAS negatively correlates with WE (Molino et al., 2022). As such we expect some differences between the measurements in the meta-regression. Since we expect certain differences in prevalences of workaholism based on sample size, sample representativeness, and measurement our study can be classified as predictive. We therefore developed three distinct hypotheses:

*Hypothesis 1*: The prevalence of workaholism will be negatively associated with sample size as small samples often are more “clinical and pathological” whereas large samples tend to provide more moderate and accurate estimates.

*Hypothesis 2*: Representativeness of the samples will moderate the prevalence estimates of workaholism in the meta-analysis in such a way that studies with representative samples will yield lower prevalence estimates than studies based on non-representative samples.

*Hypothesis 3*: The choice of instrument used to measure workaholism will significantly impact the prevalence estimates in the meta-analysis. Different assessment tools and instruments may capture different aspects of workaholism, leading to variations in prevalence rates across studies. We expect studies using more clinical instruments to report lower prevalence estimates of workaholism compared to studies using less clinical measures.

## Materials and methods

### Search strategy and inclusion criteria

**Figure 1:**
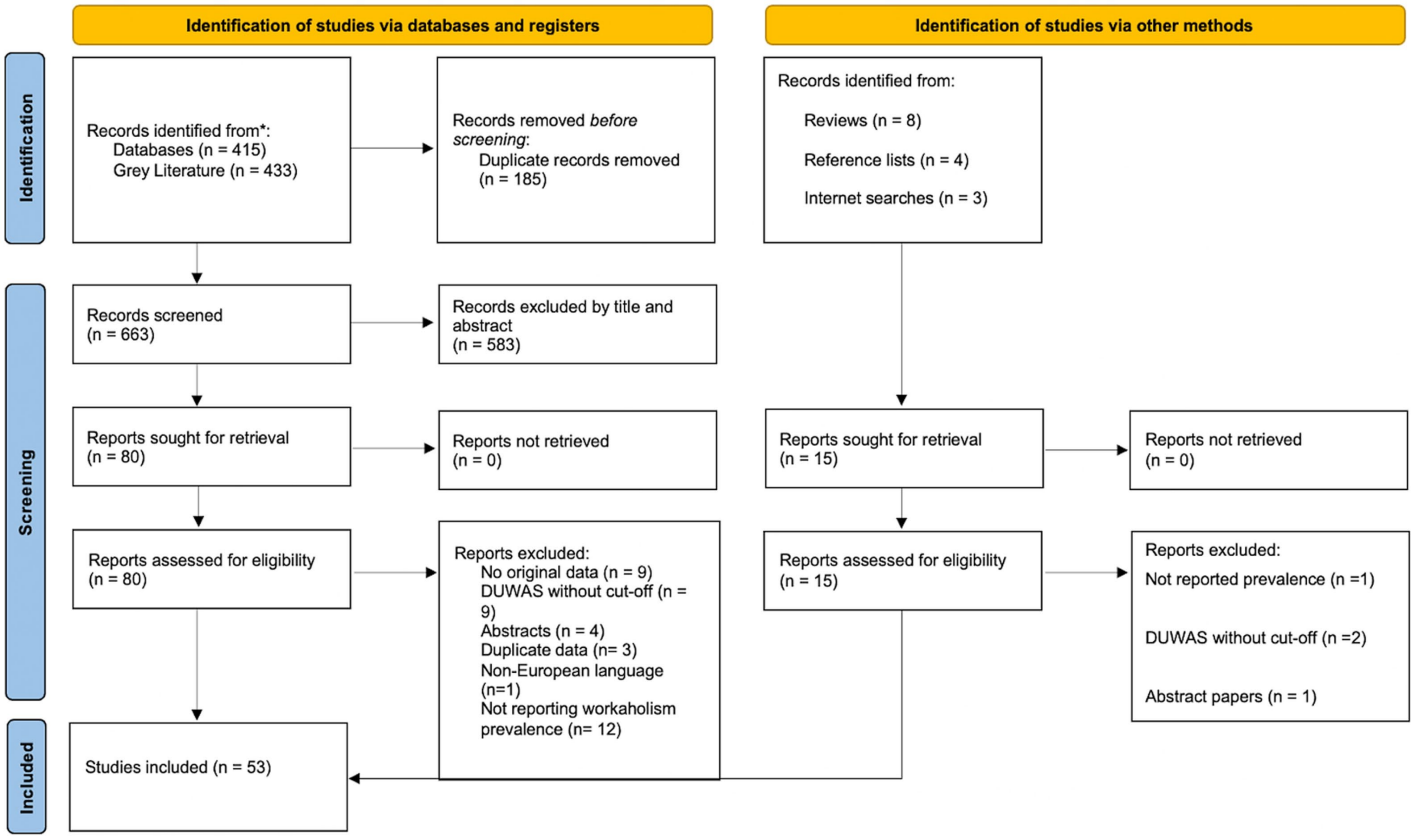
Flow diagram of systematic literature search on workaholism prevalence.

The present meta-analysis was pre-registered in the international prospective registry of systematic reviews (PROSPERO; CRD42023395794). The following search string was used: “(workaholi* OR “work addict*”) AND (prevalence* OR incident* OR frequen* OR cut-off OR epidem*).” The search was conducted in the following databases: Web of Science, PubMed, Cinahl, Embase, PsychInfo. Gray literature was searched in BASE, MedNar, NYAM, OPENGREY and OpenMD. The 200 first searches from Google Scholar were also included in the latter search. Articles were additionally searched by inspecting multiple reference lists from relevant literature. In line with the PRISMA-guidelines (Page et al., 2021) the search was supervised by a professional librarian. All identified articles were entered into EndNote. Following removal of duplicates two of the authors (VSS and MD) went manually through the remaining articles independently, applying the inclusion and exclusion criteria as guidance. The search string on databases and gray literature sources yielded 663 articles after eliminating duplicates, which were subsequently available for screening. Moreover, we identified 15 additional papers for full-text screening through other methods than a formal search. The inclusion criteria were studies reporting the prevalence of workaholism, written in any European language and based on original data. When a study was written in a language not understood by the present authors, we employed translation software (e.g., Google translate) or proactively reached out to the respective authors for necessary information. The following exclusion criteria were used based on recommendations from Taylor et al. (2014): Conference abstracts, studies based on secondary data, studies with purposive sampling of workaholics, qualitative research, and studies where cut-off for workaholism were based on the distribution (e.g., a certain percentile) rather than a clinically derived cut-off. In terms of exclusion and inclusion of studies the two raters achieved an initial agreement of 92.4%. Disagreements were resolved through discussions. No time frame was used for the literature search. The search was completed on March 2nd, 2023. The Preferred Reporting Items for Systematic Reviews and Meta-Analysis (PRISMA) was used to thoroughly report our process of literature selection (Page et al., 2021). See Figure 1 for the PRISMA flow diagram of the systematic search. All checklists related to PRISMA can be found in the Supplementary material.

### Data extraction

The relevant articles were collected, and applicable data was independently coded into an extraction form by two raters. The following data was extracted: Author(s), title, publication date, article type, description of sample (e.g., representative or non-representative and type of workers), gender, age range, mean age and standard deviation, country of study, sample size, workaholism prevalence, instrument used for assessing workaholism including reported reliability, response rate and name of journal. Most of the excluded studies were left out due to not reporting a prevalence or not reporting original data (e.g., Sussman et al., 2011; Andreassen, 2013; Aldahadha, 2019; Özsoy, 2019). Some articles that initially were thought to be included were later excluded due to not reporting a prevalence based on clear cut-offs, like Taris et al. (2012). Furthermore, one article was excluded due to using median splits in order to create groups, which makes the prevalence estimates artificial (e.g., Schaufeli et al., 2009). Further information about excluded reports is provided in the flow chart depicted in Figure 1.

A risk-of-bias evaluation of all included studies was independently conducted by two authors based on the checklist by Hoy et al. (2012) which comprises items reflecting 10 characteristics of the included studies, each scored 0 (no risk of bias) or 1 (risk of bias). Risk of bias was indicated by each of the following items: (1) study target population is not representative of the national population, (2) sampling frame is not a representation of the target population, (3) random selection is not used, (4) response rate is <75%, (5) data are collected from a proxy, (6) an acceptable case definition is not used, (7) the study instrument is not shown to have reliability or validity, (8) same mode of data collection is not used for all subjects, (9) the shortest prevalence period for the parameter is not appropriate, and (10) one or more of the numerator(s) or denominator(s) is inappropriate. Hence, the total score ranged from 0 to 10 and was categorized as follows: high quality/low risk (0 to 3), moderate quality/risk (4 to 6), and low quality/high risk (7–10; see Table 1). If synthesized data was missing in a given section of the study, it was reported as a risk. Discrepancies in terms of risk-of-bias evaluation between raters were resolved through discussions.

**Table 1:** Risk of bias/methodological quality (Hoy et al., 2012) of included studies.

References	1. *N* representative-ness	2. N-frame	3. Randomization	4. Non-response bias	5. Primary data	6. Operationalization	7. Instrument	8. Consistency	9. Period	10. Estimation	Total risk score	Risk category
Adolfo et al. (2022)	1	1	1	1	0	0	0	0	0	0	4	Moderate
Allam et al. (2021)	1	1	1	1	0	0	0	0	0	0	4	Moderate
Almeida et al. (2020)	1	1	1	1	0	1	0	0	0	0	5	Moderate
Andreassen et al. (2014)	0	0	0	0	0	0	0	0	0	0	0	Low
Andreassen et al. (2016)	1	1	1	0	0	0	0	0	0	0	3	Low
Ariapooran (2019)	1	0	0	0	0	0	0	0	0	0	1	Low
Atroszko et al. (2017)	1	1	1	1	0	0	0	0	0	0	4	Low
Azevedo and Mathias (2017)	1	0	0	0	0	1	0	0	0	0	2	Low
Bellali et al. (2022)	1	1	1	1	0	0	0	0	0	0	4	Moderate
Berthelot et al. (1999)	1	0	0	0	0	1	1	0	0	0	3	Low
Borbala et al. (2021)	0	0	0	0	0	1	0	0	0	0	1	Low
Borbala et al. (2017)	0	0	0	0	0	1	0	0	0	0	1	Low
Borbála et al. (2009)	0	0	0	0	0	1	0	0	0	0	1	Low
Borges et al. (2021)	1	1	1	1	0	1	0	0	0	0	5	Moderate
Burke and Matthiesen (2004)	1	0	0	0	0	0	0	0	0	0	0	Low
Burke (1999)	1	1	0	1	0	0	0	0	0	0	3	Moderate
Burke et al. (2004)	1	0	1	1	0	0	0	0	0	0	3	Low
Demetrovics et al. (2022)	0	0	0	0	0	1	0	0	0	0	1	Low
Doerfler and Kammer (1986)	1	1	1	1	0	0	1	0	1	0	6	Moderate
Eason et al. (2022)	1	1	1	1	0	0	0	0	0	0	4	Moderate
Galdino et al. (2021)	1	1	1	1	0	0	0	0	0	0	4	Moderate
Garcia et al. (2022)	1	1	1	0	0	0	0	0	0	0	3	Low
Hogan et al. (2016)	1	1	1	1	0	0	0	0	0	0	4	Moderate
Hrairi et al. (2022)	1	1	1	1	0	0	0	0	0	0	4	Moderate
Kanai et al. (1996)	1	1	1	0	0	0	0	0	0	0	3	Low
Kang (2020)	0	0	0	0	0	0	1	0	0	0	1	Low
Kasemy et al. (2020)	1	0	0	0	0	0	0	0	0	0	1	Low
Kilroy (2007)	1	1	0	1	0	0	0	0	0	0	3	Low
Kubota et al. (2010)	1	1	1	1	0	1	0	0	0	0	5	Moderate
Kun et al. (2020a)	0	0	0	0	0	0	0	0	0	0	0	Low
Kunecka and Hundert (2018)	1	0	0	0	0	0	1	0	0	0	2	Low
Lichtenstein et al. (2019)	1	1	1	1	0	0	0	0	0	0	4	Moderate
Loscalzo et al. (2022)	1	1	1	1	0	0	1	0	0	0	5	Moderate
Marmet et al. (2019)	1	0	0	0	0	1	0	0	0	0	2	Low
Moaouad et al. (2012)	1	1	1	1	0	1	1	0	0	0	6	Moderate
Molino et al. (2022)	1	1	1	1	0	0	0	0	0	0	4	Moderate
Nazligul et al. (2021)	1	1	1	1	0	0	0	0	0	0	4	Moderate
Nunes (2000)	1	0	1	1	0	0	0	0	0	0	3	Low
Orosz et al. (2016)	0	0	0	0	0	0	0	0	0	0	0	Low
Park et al. (2020)	0	0	0	0	0	0	1	0	0	0	1	Low
Ravoux et al. (2018)	1	1	1	1	0	0	0	0	0	0	4	Moderate
Rezvani et al. (2014)	1	0	1	1	0	0	0	0	0	0	3	Low
Rogowska et al. (2021)	1	1	1	1	0	0	0	0	0	0	4	Moderate
Romdhane et al. (2022)	1	1	1	1	0	0	0	0	0	0	4	Moderate
Sheta and Hammouda (2022)	1	1	1	1	0	0	0	0	0	0	4	Moderate
Shimazu et al. (2011)	1	1	1	1	0	0	0	0	0	0	4	Moderate
Spence and Robbins (1992)	1	1	1	1	0	0	0	0	0	0	4	Moderate
Sussman et al. (2015)	1	1	1	1	0	1	1	0	1	0	7	High
Thege et al. (2014)	1	1	0	0	0	1	1	0	0	0	5	Moderate
Urban et al. (2019)	0	0	0	0	0	0	0	0	0	0	0	Low
Villella et al. (2010)	1	0	0	0	0	0	0	0	0	0	1	Low
Winburn et al. (2017)	1	1	1	1	0	0	0	0	0	0	4	Moderate
Ziemska et al. (2013)	1	1	1	1	0	1	1	0	0	0	6	Moderate

Score: (0: low risk, 1; high risk); Total risk score: [range (0–10): low risk (0–3), moderate risk (4–6), high risk (7–10)]. N frame = sampling frame.

### Statistical analysis

We employed a random-effects model in our meta-analysis to account for the fact that the individual studies represented different populations (Borenstein et al., 2021). The DerSimonian and Laird (1986) approach was used to estimate the between-study variance. Prevalence estimates and their corresponding 95% confidence intervals (CIs) were calculated. If there was significant heterogeneity between studies, a random-effects meta-regression analysis was conducted to explore the following *a priori* determined predictors of the dispersion of prevalences: (a) sample size, (b) representativeness (representative vs. non-representative), and (c) workaholism instruments (where BWAS constituted the contrast). We assessed heterogeneity using Cochran’s Q test and calculated the I^2^-statistic, which indicates the proportion of variation in observed effects that is due to true effects (Borenstein et al., 2017). An I^2^-value of 0% suggests no heterogeneity, while values of 25, 50, and 75% indicate low, moderate, and high heterogeneity, respectively (Higgins et al., 2003). Additionally, we calculated the 95% prediction interval, which represents the range in which the effect size of a future study would likely fall into if randomly selected from the same population as the studies in the present meta-analysis (IntHout et al., 2016). To investigate publication bias, we utilized the trim-and-fill procedure developed by Duval and Tweedie (2000). This procedure examines the funnel plot, where effect sizes are plotted against the inverse of the variance (sample size). A symmetrical funnel plot indicates no publication bias, while an asymmetrical plot usually suggests a lack of small studies with small effects. The trim-and-fill procedure identifies and adjusts for asymmetric outlying studies, providing an adjusted effect size and 95% CI. The meta-analysis was conducted using the Comprehensive Meta-Analysis, version 4 (Borenstein, 2022). The software employed logit transformation of the data to calculate prevalences, which then were back-transformed to their original metric. As for sensitivity analysis, separate meta-analyses were conducted for certain non-working samples, and representative and nationally representative samples.

## Results

### Description of studies

Information regarding the authors of the included studies can be found in Table 2. Of the 53 included studies, publication years ranged from 1986 (k = 1) to 2022 (k = 7). Studies were conducted in Hungary (k = 7), the United States (k = 7), Norway (k = 4), Brazil (k = 3), Egypt (k = 3), France (k = 3), Japan (k = 3), Poland (k = 3), Canada (k = 2), Italy (k = 2), South Korea (k = 2), Lebanon (k = 2), and one study from each of the following countries: Denmark, Greece, Iran, Ireland, Israel, Portugal, Saudi-Arabia, Spain, Switzerland, Tunisia, and Turkey.

**Table 2:** Characteristics of studies and continuation of the table.

References	Country	Description of sample	Measurement	*n*	*n* (Female)	*n* (male)	Age range	Age (M and SD) ±	Prevalence %	RR %
Adolfo et al. (2022)	Saudi-Arabia	Nurses	WART	427	124	303	23–61	33.82 ± 4.94	37.0	
Allam et al. (2021)	Egypt	University staff	DUWAS	336	172	164	22–63		33	
Almeida et al. (2020)	Brazil	Graduate nursing professors	DUWAS	333					10.5	
Andreassen et al. (2014)	Norway	Norwegian workers	BWAS	1,124			18–70		8.3	54
Andreassen et al. (2016)	Norway	Norwegian workers	BWAS	16,426	10,487	5,939	16–75	37.3 ± 11.4	7.8	
Ariapooran (2019)	Iran	Nurses	DUWAS	247				31.03 ± 5.44	13.77	86.4
Atroszko et al. (2017)	Poland	Various polish professions	BWAS	723	513	200	20–79	±11.33	17.4	
Azevedo and Mathias (2017)	Brazil	Physicians	DUWAS	1,108	512	591		44.42 ± 13.89	44.9	
Bellali et al. (2022)	Greece	Health professionals	BWAS	542	429	113		43.30	18.5	87.6
Berthelot et al. (1999)	France	Rheumatology patients	OTHER	125	61	64			21	
Borbala et al. (2021)	Hungary	National representative study	BWAS	1,324			18–64		5,1	
Borbala et al. (2017)	Hungary	National representative study	BWAS	1,315			18–64		4,7	
Borbála et al. (2009)	Hungary	National representative study	WART	2,439					5.7	
Borges et al. (2021)	Portugal	Nurses	DUWAS	839	688	151			27.1	
Burke and Matthiesen (2004)	Norway	Journalists	WorkBAT	211	70	141			9.5	
Burke (1999)	Canada	MBA Graduates	WorkBAT	530	530				13.2	35
Burke et al. (2004)	Norway	Senior managers	WorkBAT	171	1	170	36–45		12	13
Demetrovics et al. (2022)	Hungary	General population	WART	2,710	1,382	1,328		39.8 ± 13.6	1.5	
Doerfler and Kammer (1986)	United States	Attorneys, physicians, and psychologists	OTHER	192	106	86			23	53
Eason et al. (2022)	Not reported	Athletic Trainers	WART	226	161	65	22–63	32 ± 9	23	
Galdino et al. (2021)	Brazil	Professors		368	311	57	28–75	53.00	35.5	40.1
Garcia et al. (2022)	Spain	Nurses	DUWAS	219	199	20		40.9 ± 10.6	28.3	76.9
Hogan et al. (2016)	Ireland	Academics	WorkBAT	410	204	206			27	27
Hrairi et al. (2022)	Tunisia	Engineers	WART	107	45	62		29.2 ± 4.4 years	42.1	
Kanai et al. (1996)	Japan	Industrial workers	OTHER	1,072	110	962		M (male) = 42.3 M (female) = 28.0	18.75	87.5
Kang (2020)	South Korea	Workers	OTHER	4,242	1,745	2,497	20–69	39.0	39.7	
Kasemy et al. (2020)	Egypt	Health care workers and non-health care workers	DUWAS	1,126					15.2	
Kilroy (2007)	United States	Managers and non-managers	WorkBAT	58	16	42			32.8	52.7
Kubota et al. (2010)	Japan	Nurses	DUWAS	312	312		21–60	30.9 ± 7.5	28.53	65.7
Kun et al. (2020a)	Hungary	Hungarian	BWAS	1,490			16–64		8	
Kunecka and Hundert (2018)	Poland	Nurses	OTHER	975	951	24			5.95	
Lichtenstein et al. (2019)	Denmark	Random workers through online advertisement	BWAS	671	514	157	16–68	40.1	6.6	
Loscalzo et al. (2022)	Israel	Workers	W-10	459	253	206	19–69	37.12 ± 10.33	8.7	
Marmet et al. (2019)	Switzerland	Young men	BWAS	5,516		5,516		19.97 (SD = 1.22) years old at baseline and 25.47 (SD = 1.26)	8.1	
Moaouad et al. (2012)	Lebanon	Medical students	OTHER	280	148	132	17–24		20.4	
Molino et al. (2022)	Italy	Volunteers	BWAS	1,035	304	283	20–66	41.31 ± 11.31	7.6	
Nazligul et al. (2021)	Turkey	Workers	BWAS	448	279	169		38.75 ± 9.92	18.1	
Nunes (2000)	United States	Park and recreational professionals	WART	258	99	159	22–73	41.92	14	
Orosz et al. (2016)	Hungary	National representative study	BWAS	500	251	249	15–59	35.05 ± 11.97	20.6	
Park et al. (2020)	South Korea	National representative study	OTHER	3,157	1,079	2,078	15 and older		6.7	
Ravoux et al. (2018)	France	Workers	WART	187	95	92		41.6 ± 11.7	20.8	11.83
Rezvani et al. (2014)	France	Physicians	WART	444			27–67	42.4 ± 10.1	13	45
Rogowska et al. (2021)	Poland	Undergraduates	WART	182	102	80	20–28	22.17 ± 1.39	13.7	
Romdhane et al. (2022)	Lebanon	Workers	BWAS	1,268	825	443		26.18 ± 11.17	13.8	
Sheta and Hammouda (2022)	Egypt	Physicians	WART	262	127	135			14.5	
Shimazu et al. (2011)	Japan	Workers	DUWAS	1,988	944	944			29	33.4
Spence and Robbins (1992)	United States	Social workers	WorkBAT	291	157	134			11	49.0
Sussman et al. (2015)	United States	Workers	OTHER	538	279	259		19.9 ± 0.85	19.5	75.0
Thege et al. (2014)	Canada	Workers	OTHER	6,000			18 and older		17.23	78.0
Urban et al. (2019)	Hungary	National representative study	WART	2,710	1,165	1,545	18–64	38.9 ± 10.8	9.3	85.1
Villella et al. (2010)	Italy	Students	WART	2,853	1,145	1,711	13–20	17.7 ± 1.9	7.6	87.8
Winburn et al. (2017)	United States	School counselors	BWAS	341	289	40			14.65	28.0
Ziemska et al. (2013)	Poland	Health professionals	OTHER	2,486	1,602	884			29.45	

WART, Work Addiction Risk Test; DUWAS, Dutch Work Addiction Scale; BWAS, Bergen Work Addiction Scale; WorkBAT, the Workaholism Battery; RR, response rate.

A minority of the 53 included studies were representative (k = 16; Burke and Matthiesen, 2004; Borbála et al., 2009; Andreassen et al., 2014; Orosz et al., 2016; Azevedo and Mathias, 2017; Borbala et al., 2017, 2021; Kunecka and Hundert, 2018; Ariapooran, 2019; Marmet et al., 2019; Urban et al., 2019; Kang, 2020; Kasemy et al., 2020; Kun et al., 2020a; Park et al., 2020; Demetrovics et al., 2022), and nationally representative (k = 10; Borbála et al., 2009; Andreassen et al., 2014; Orosz et al., 2016; Borbala et al., 2017, 2021; Urban et al., 2019; Kang, 2020; Kun et al., 2020a; Park et al., 2020; Demetrovics et al., 2022).

A majority of the studies used BWAS in order to assess workaholism (k = 14). Some were based on the WART (k = 12), whereas a minority of the studies employed measurements coded as others, e.g., WAQ, K-WAQ (k = 11), DUWAS (k = 10), and WorkBAT (k = 6). More information on which instrument that was employed in the different studies can be found in Table 2.

Samples majorly consisted of professions which varied from various workers (k = 12), nurses (k = 6), other health professionals, e.g., physicians (k = 5). Four studies consisted of samples with a combination of specific professions and two studies assessed workaholism in professors. Other studies used samples of academics (k = 1), athletic trainers (k = 1), engineers (k = 1), industrial workers (k = 1), journalists (k = 1), managers (k = 1), MBA graduates (k = 1), parks and recreational workers (k = 1), school counselors (k = 1), social workers (k = 1), university staff (k = 1), volunteers (k = 1), conscripts/soldiers (k = 1), and some samples consisted of populations that may have included non-working participants such as the Hungarian population (k = 7), students (k = 3) and patients (k = 1). Please see Table 2 for more details.

In total, the studies included 71,625 participants, with samples ranging from 58 (Kilroy, 2007) to 16,426 (Andreassen et al., 2016). The mean sample size was 1351.6 (SD = 2472.2). Overall, the studies had a fairly equal sex balance, 28,786 females and 28,401 males. Table 2 presents a detailed overview of the study characteristics of the included studies.

### Prevalence estimates and heterogeneity

The results of the meta-analysis are presented in Figure 1. The overall workaholism prevalence across all 53 studies was 15.2% (95% CI = 12.4.–18.5). Cochran *Q* was significant (*Q* = 5461.7, *df* = 52, *p* < 0.000), suggesting heterogeneity across the prevalence estimates, and the *I*^2^ statistic was 99.1%, indicating very high heterogeneity. The 95% prediction interval was 3.0–51.0%.

### Sensitivity analysis

When calculating the prevalence based solely on representative studies the overall prevalence was reduced to 9.8% (95% CI = 5.7–16.3). Furthermore, when only accounting for nationally representative studies the overall prevalence was even smaller; 8.0% (95% CI = 3.4–17.8). Lastly, a separate analysis was conducted with the samples consisting of patients and students. The analysis of these samples resulted in a prevalence rate of 14.6% (95% CI = 7.9–24.5%) which did not deviate much from the overall pooled estimate.

### Correlates of workaholism prevalence

Because of the significant heterogeneity, a meta-regression analysis based on a random-effects model was conducted including sample size, workaholism measurement (which was dummy coded including WART, WorkBAT, DUWAS, and other instruments with BWAS constituting the reference category), and representativeness (non-representative = 0, representative = 1) as moderators. The results are presented in Table 3. Overall, the regression model was found to be significant (*Q* = 23.1, *df* = 6, *p* = 0.008, *R*^2^ = 0.29).

### Publication bias

The trim-and-fill procedure trimmed three studies and changed the overall prevalence to 14.1, 95% CI = 11.2–17.6 (Q = 8418.4). The three imputed studies reflect a counterpart to the three studies with the highest prevalences (Azevedo and Mathias, 2017; Kang, 2020; Hrairi et al., 2022).

### Interrater reliability

Interrater reliability in terms of percent agreement was calculated separately for three specific coding processes: Article inclusion and exclusion (by VVS and MD), coding of study characteristics (by FA and MD) and risk of bias evaluation (by FA and MD) using the Hoy et al. (2012) framework. The calculated percentages were 92.4%, 84.9%, and 87.0%, respectively. Disagreements were resolved through discussions.

### Summary of findings

A total of 53 studies fulfilled the inclusion criteria, thus being included in the meta-analysis. Consequently, the present study indicates an overall workaholism prevalence of 15.2%, which was adjusted to 14.1% following a trim-and-fill adjustment for publication bias. The dispersion of effect sizes between studies were significant, ranging from 1.5% (Demetrovics et al., 2022) to 44.9% (Azevedo and Mathias, 2017). The present study’s estimated prevalence suggests that roughly 1 in 7 might be affected with workaholism. This indicates that workaholism is a prevalent issue which is a concern as it is well known that workaholism takes a heavy toll on people’s health and wellbeing (Robinson et al., 2006; Schaufeli et al., 2008a Andreassen et al., 2013a 2016, 2018a Haar and Roche, 2013; Matsudaira et al., 2013; Shimazu et al., 2015; Clark et al., 2016; Salanova et al., 2016 Yang et al., 2020; Balducci et al., 2021 Galdino et al., 2021 Chang et al., 2022; Cheng and Gu, 2022 Sarfaraz et al., 2022).

## Discussion

The present study is the first meta-analysis concentrated on the prevalence of workaholism. The vast majority of the included studies employed single sample designs, with each study focusing on the prevalence in a specific population. Due to the variation in measurement tools, operationalization, sampling methods and other methodological factors the included studies report conflicting and inconsistent findings, which was clearly shown by the high and significant heterogeneity. Based on the results of the overall prevalence (15.2%) workaholism seems to affect a relatively high percentage of people, suggesting that this behavioral addiction is generally common. Future studies should therefore focus on prevention and treatment of workaholism, considering there is wide scientific support attesting to the health risks associated with workaholism.

In terms of the risk of bias analysis, showed in Table 1, about half of the studies were deemed to have a moderate risk of bias. Some of the factors contributing to risk of bias was the usage of convenient samples, e.g., recruiting participants online for example through links on social media, and reporting a low response rate (or not reporting this at all). There was also a frequent issue with randomization in many studies. Based on the Hoy et al. (2012) checklist, we only identified one study displaying an overall high risk score, whereas 26 studies had a moderate total risk score, and the remaining 26 a low total risk score. Most of the included studies were cross-sectional but there were a few exceptions as some studies followed-up on participants at a later time (e.g., 1 year; Sussman et al., 2015). Longitudinal studies offer advantages by assessing the stability of workaholic tendencies as well as possibly identifying real antecedents of workaholism.

The regression model was found to be overall significant, but sample size did not significantly moderate the prevalence of workaholism and thus our first hypothesis was not supported. In contrast the second hypothesis which stated that representative samples would yield lower prevalence estimates of workaholism was found to be supported, implying that non-representative studies are associated with higher prevalences than studies based on representative samples. Non-representative samples are often more prone to sampling biases (e.g., self-selection) which might artificially inflate the estimates (Riffenburgh, 2012). Studies that were coded as representative consisted of samples from groups that accurately represents the characteristics of the target population (e.g., nurses; Kunecka and Hundert, 2018). Other studies that employed national surveys which included a large group that represents the characteristics of the national population and/or national worker population were coded as nationally representative (e.g., Andreassen et al., 2014; Kang, 2020). To further solidify the assumption that representativeness was associated with the prevalence of workaholism we conducted two separate sensitivity analyses excluding all non-representative studies. In total 16 out of 53 studies were coded as representative. These had a pooled prevalence of 9.8%, which suggests that the overall pooled prevalence of 15.3% might be an overestimate. The second sensitivity analysis excluded all studies that were not representative of either a national population or national working population. This reduced the overall prevalence of workaholism to 8.0% supporting hypothesis 2. For a few of the samples (e.g., Borbála et al., 2009; Borbala et al., 2017, 2021) there were some uncertainties as to whether all were workers. However, as non-workers are expected to score low on workaholism measures, including these samples more likely lead to an underestimation rather than an overestimation of the workaholism prevalence. Still, this should be taken into account when interpreting the findings. Nonetheless, the majority of the included studies consisted of working samples and consequently the reported prevalence provides valuable insight into the number of workaholics in the working population (Table 3).

**Table 3:** Results of meta-regression of representativeness, instruments, and sample size on workaholism prevalence.

Predictor	Coefficient	SE	95% CI	Z	2-sided *p*
Intercept	1.782	0.239	−2.252 to −1.313	−7.44	0.000
Representativeness	−0.651	0.227	−1.096 to −0.205	−2.87	0.004
**Instruments**					
Other^1^	0.453	0.302	−0.139 to −1.045	1.50	0.133
WART^1^	0.115	0.300	−0.473 to −0.704	0.39	0.700
WorkBAT^1^	0.244	0.382	−0.505 to −0.994	0.64	0.522
DUWAS^1^	0.923	0.316	0.303 to 1.543	2.92	0.003
Sample size	−0.000	0.000	−0.0001 to 0.000	−0.99	0.323

Representativeness criteria (representative = 1, non-representative = 0); ^1^BWAS is the contrast.

As stated in hypothesis 3 we assumed that studies using more clinical instruments would report lower prevalence estimates of workaholism compared to studies using less clinical measures. In support of this presumption DUWAS was found to produce higher prevalence rates than BWAS. The reason for this is most likely that the BWAS was constructed based on clinical symptoms of addiction (Andreassen et al., 2012) whereas DUWAS was based on factor analyses of items from the WART (Schaufeli et al., 2009) and the drive subscale of the WorkBat (Schaufeli et al., 2009). The WART was originally based on a list of symptoms reported by clinicians who were involved in diagnosing workaholism whereas the drive subscale of the WorkBat originated from a theoretical understanding of workaholism proposed by its authors. Although the WART and the WorkBat are linked to clinical notions about workaholism, their items are not rooted in an overarching clinical model to the same degree as the BWAS which was based on a comprehensive component model of addiction (Andreassen et al., 2012), hence making the DUWAS less anchored in a clinical psychopathological framework.

### Implications of the overall prevalence

The overall prevalence of workaholism can be described as high and since it has been speculated that workaholism may be on the rise (Andreassen et al., 2016), we recommend that future meta-analyses on this topic include time as a moderating variable. Secondly, as the present study included a minority of studies that may be non-working future meta-analyses could opt to only include working populations to better represent the prevalence of workaholism. Lastly, we recommend for future research to examine the effectiveness of interventions to prevent and treat workaholism, both at the individual and organizational level. So far, several promising treatments have been identified (Van Wĳhe et al., 2010; Van Gordon et al., 2017). In order to spur proactive action against workaholism, we believe that recognizing workaholism as a serious behavioral addiction will facilitate the development of treatments and combative strategies. To provide context for this proposal, gambling disorder and gaming disorder have been deemed as serious behavioral addictions and are currently recognized as such in current psychiatric nosology (American Psychiatric Association, 2013; World Health Organization, 2019) whereas workaholism has yet to be formally regarded as a behavioral addiction (Andreassen, 2013; Loscalzo and Giannini, 2017). This indicates that workaholism as a societal problem is overlooked. One possible reason for the lack of action regarding workaholism is that it seems to bear short-term fruits for the organizations, and has as such, in a similar vein as exercise addiction, been denoted as a “productive” and “positive addiction” (Andreassen et al., 2013a). In line with such a perspective, excessive work is often positively sanctioned with praise and rewards, compared to gambling and gaming addiction which generally are considered to reflect “stupidity” and “laziness” (Miller and Thomas, 2017; Casale et al., 2023).

### Implications of the moderators

Based on the results from the meta-regression future studies could benefit from validating existing instruments in cross-cultural samples and conducting comparative assessments of the popular measurements. Two studies based on nationally representative samples of Korean workers reported significantly different prevalence rates, ranging from 6.7% (Park et al., 2020) to 39.7% (Kang, 2020). Since the two samples share major similarities, the disparity between the reported workaholism prevalences is unanticipated. A potential explanation of the prevalence disparity may be linked to the utilization of different workaholism measures. Park et al. (2020) used the Workaholism Analysis Questionnaire (WAQ) and Kang (2020) developed a Korean form of the WAQ (K-WAQ). Since the K-WAQ was specifically created with Korean workers in mind it may be more suited for capturing workaholism in Korea (Kang, 2020). Similar differences may apply to other measurements of workaholism in given cultures and countries. Certain instruments which were used by some of the included studies have not been as extensively psychometrically evaluated (e.g., WAQ) in contrast to more prominent instruments (e.g., DUWAS, BWAS; Andreassen, 2013).

In order to produce comparable findings in workaholism research it is necessary for measurements to be more thoroughly validated against each other. Since most prominent measurements on workaholism are based on Western samples, e.g., BWAS (Andreassen et al., 2012) it would be worthwhile to investigate their applicability for detecting workaholism in Asian cultures. Testing these instruments and validating them in cross-cultural samples is an important scientific endeavor and a core issue that needs to be addressed by future studies. We thus recommend researchers to explore the validity and usefulness of the BWAS in non-European samples (e.g., Egypt, Japan and South Korea). Lastly, the results showed a significant difference between DUWAS and BWAS in terms of prevalence estimates even though some scholars categorize them as similar in terms of measuring work addiction (Hu, 2018). Since the present findings suggest that their threshold for categorization of workaholism is different we believe it would be worthwhile to conduct more stringent empirical comparison of the two instruments. Future research could also consider technology-based approaches, for example ecological momentary assessment (Office for Health Improvement and Disparities, 2020), to achieve measurement of workaholism in real time.

In terms of hypothesis 2 the present study highlights the tendency for non-representative samples to yield higher prevalence estimates of workaholism. If workaholism is to be prevented and intervened against it is vital that studies properly represent target populations so that stakeholders and organizations can understand the severity of workaholism and allocate adequate resources toward reducing the overall rate of workaholics. We therefore recommend future studies to prioritize resources toward the conduction of workaholism research with representative samples of target populations. In doing so researchers can strengthen the generalizability of their findings and reduce the inherent problems arising from comparing studies with different samples.

### Theoretical strengths

Firstly, the present study extends prior theoretical work by providing a clear pooled estimate of workaholism. Secondly, our regression model was overall significant and as we expected some instruments report higher frequencies of workaholism compared to more addiction-based measurements (e.g., BWAS). Lastly, we consider the discovered moderating effect of representativeness as another theoretical strength of the results as it establishes evidence which indicate that non-representative studies yield higher prevalences of workaholism. By highlighting this effect, the present study supports other researcher’s assumptions (e.g., Andreassen, 2013) about the need for more representative samples in studies assessing the prevalence of workaholism.

### Methodological strengths

To strengthen this meta-analysis’s findings, we included gray literature and employed several methodological procedures to ensure reliability. The inclusion of gray literature is recommended for obtaining unbiased estimates (Borenstein et al., 2021) and reducing the possibility of publication bias as well as publication time lag. Next, all the prevalence data and quality assessments were independently coded by two authors. This diminishes the risk for overlooking important information. Furthermore, we evaluated the agreement for inclusion/exclusion, information extraction, and risk of bias/study quality. This strategy benefits the study by fostering a data discussion platform, incorporating varied viewpoints, and facilitating the consideration of possibly overlooked studies. We also kept records of initial disagreements between raters which made it possible to calculate the interrater reliability. This meta-analysis also benefited from our diligent efforts to find relevant studies beyond electronic searches. We systematically explored reference lists from original and review papers, adding 11 studies to the analysis. Hence, we believe our meta-analysis provides a comprehensive coverage of literature based on European languages. Furthermore, as in accordance with the PRISMA 2020 guidelines (Page et al., 2021), our meta-analysis included proactively contacting original authors for missing study information relevant to our analyses. Lastly, without a timeframe exclusion criterion, we ensured wide-ranging literature coverage.

### Theoretical limitations

While our meta-analysis offers valuable insights, it is limited by certain theoretical factors, such as the conceptual ambiguity of workaholism, the inclusion of possible non-working samples and the exclusion of culture and time as moderators. We conceptualized “workaholism” and “work addiction” as equivalent although some scholars separate the two (Morkevičiūtė and Endriulaitienė, 2022). Secondly, as the present study omitted country/national culture as a moderator of workaholism we may have failed to account for a part of the unexplained variance of workaholism prevalences. In this regard it should be noted that some studies have indicated that certain Asian cultures are more prone to workaholic tendencies and therefore more likely report higher frequencies of workaholism (Krumov et al., 2022). Subsequently, in the present study samples based in Asian countries (e.g., Japan) seemingly reported higher prevalences of workaholism compared to European counterparts (e.g., Kubota et al., 2010; Shimazu et al., 2011; see Table 2). Still, some of these high rates of workaholism in Asian samples may be partially explained by the preferred usage of the DUWAS and other instruments (e.g., K-WAQ). Lastly, over time, the understanding of workaholism has endured substantial changes which suggests that early research could have captured prevalence rates that are not as applicable for comparison with newer reported rates of workaholism.

### Methodological limitations

**Figure 2:**
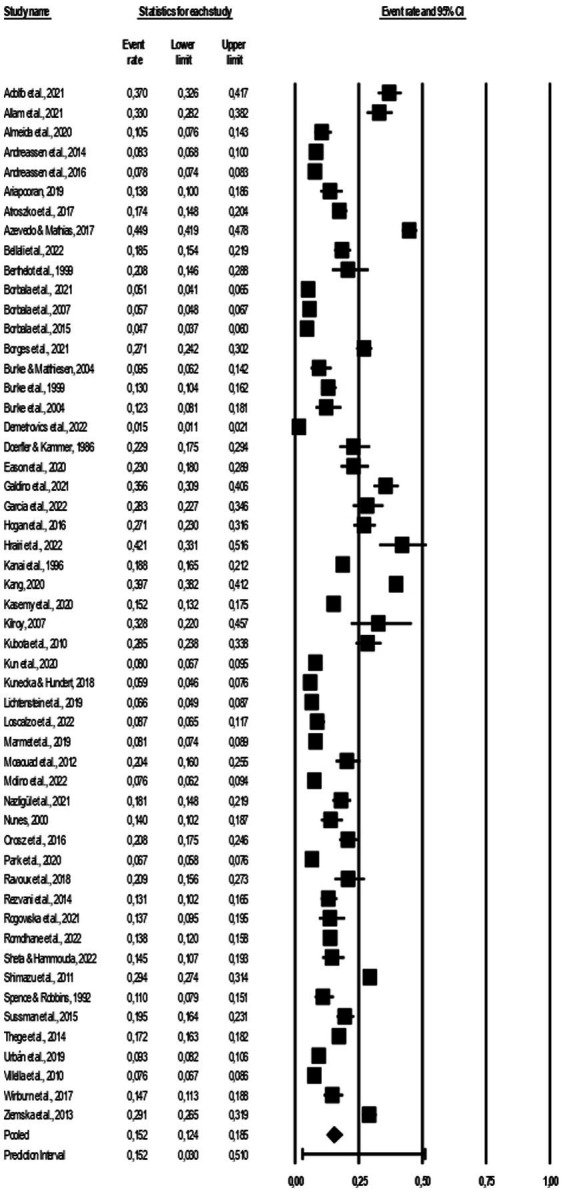
Forest plot of the included studies.

Our meta-analysis has certain methodological limitations due to the data quality of the included studies and the employed search string. Our analysis included studies of varying quality/risk of bias, consequently, the findings could have been influenced by other methodological factors, in addition to the *a priori* determined moderators. A large portion of the included studies had a moderate risk of bias owed to issues with randomizations and convenient sampling which weakens the validity and reliability of the reported prevalences (Figure 2).

We cannot rule out that some relevant papers have been overlooked. Some of the included papers were identified through other means (e.g., backward tracking) which may imply that the search string employed had too low sensitivity. Additionally, the present study only included articles written in European languages due to the authors language proficiency and the quality of available translation software. As we restricted our search to papers written in European languages, studies in non-European languages were excluded which might have caused a bias. Lastly, by limiting the search to English words, papers written in a European language, where neither the title nor abstract were available in English, were at a significant risk of being overlooked.

## Conclusion

The present meta-analysis provides a comprehensive summary of significant and emerging research on work addiction, shedding light on a relatively new topic within the field of behavioral addictions. Moreover, it establishes an estimated prevalence rate of 15.2% and an adjusted rate of 14.1% following a trim-and-fill adjustment for publication bias which serves as a foundation for future research. The three emphasized moderators explained 29% of the variance, suggesting that these methodological constructs provide valuable insight into estimates of workaholism. Furthermore, it implies that future research should include more moderators in order to identify other factors with a moderating effect such as time, culture and sex.

Moreover, we discovered a pattern where some measurements produce higher prevalences than others and a tendency for representative samples to yield lower prevalence estimates. Thus, we emphasize the need for a more universally accepted operationalization of workaholism and recommend future studies to use representative samples. The usage of many different instruments and non-representative samples contribute to discrepancies and unsteady prevalence findings. Despite some acknowledged limitations, the present meta-analysis yields valuable insights and findings which add to and synthesizes the current literature. It is hoped that the results will prompt other researchers to accord greater priority to this phenomenon and that the current findings spur preventive and treatment initiatives as well as contribute to the formal recognition of workaholism as a behavioral addiction.

## Data availability statement

The raw data supporting the conclusions of this article will be made available by the authors, without undue reservation.

## Author contributions

Study conceptualization was conducted by SP. Literature search was conducted by VS and MD. Data analysis was conducted by FA. The coding of studies were conducted by FA and MD. FA and SP were responsible for the interpretation of the data. FA, MD, VS, and SP contributed to the writing. FA was responsible for the revisions of the article. All authors contributed to the article and approved the submitted version.
