# Supplementary material for: The prevalence of workaholism: a systematic review and meta-analysis
Source: Front Psychol. 2023 Oct 30;14:1252373. doi: 10.3389/fpsyg.2023.1252373 (PMC10643257; doi:10.3389/fpsyg.2023.1252373)
Supplement: Supplementary file 1 [file Table_1.DOCX]

Supplementary Material

## 1. Supplementary Figures

**Supplementary Figure 1**: PRISMA abstract checklist

**Supplementary Figure 2**: PRISMA 2020 checklist

**Supplementary Figure 3**: PRISMA 2020 checklist

**Supplementary Figure 4**: Funnel Plot from the meta-analysis on the prevalence of workaholism, adjusted by using Duval and Tweedis trim and fill.

**Supplementary Table 1: Search terms and strategy**

| **Searches and databases** |
| --- |
| All fields: “(workaholi* OR "work addict*") AND (prevalence* OR incident* OR frequen* OR cut-off OR epidem*)” |
| **APA PsycINFO (Ovid)** |
| “(workaholi* OR "work addict*") AND (prevalence* OR incident* OR frequen* OR cut-off OR epidem*)” |
| **EMBASE (Ovid)** |
| “(workaholi* OR "work addict*") AND (prevalence* OR incident* OR frequen* OR cut-off OR epidem*)” |
| **MEDLINE (Ovid)** |
| “(workaholi* OR "work addict*") AND (prevalence* OR incident* OR frequen* OR cut-off OR epidem*)” |
| **Web of Science** |
| “(workaholi* OR "work addict*") AND (prevalence* OR incident* OR frequen* OR cut-off OR epidem*)” |
| **PubMed** |
| “(workaholi* OR "work addict*") AND (prevalence* OR incident* OR frequen* OR cut-off OR epidem*)” |
| **CINAHL** |
| “(workaholi* OR "work addict*") AND (prevalence* OR incident* OR frequen* OR cut-off OR epidem*)” |
| **BASE** |
| “(workaholi* OR "work addict*") AND (prevalence* OR incident* OR frequen* OR cut-off OR epidem*)” |
| **MedNar** |
| “(workaholi* OR "work addict*") AND (prevalence* OR incident* OR frequen* OR cut-off OR epidem*)” |
| **NYAM** |
| “(workaholi* OR "work addict*") AND (prevalence* OR incident* OR frequen* OR cut-off OR epidem*)” |
| **OPENGREY** |
| “(workaholi* OR "work addict*") AND (prevalence* OR incident* OR frequen* OR cut-off OR epidem*)” |
| **OpenMD** |
| “(workaholi* OR "work addict*") AND (prevalence* OR incident* OR frequen* OR cut-off OR epidem*)” |
| **Google Scholar** |
| “(workaholi* OR "work addict*") AND (prevalence* OR incident* OR frequen* OR cut-off OR epidem*)” |
