# Supplementary figures and images for: The prevalence of workaholism: a systematic review and meta-analysis
Source: Front Psychol. 2023 Oct 30;14:1252373. doi: 10.3389/fpsyg.2023.1252373 (PMC10643257; doi:10.3389/fpsyg.2023.1252373)

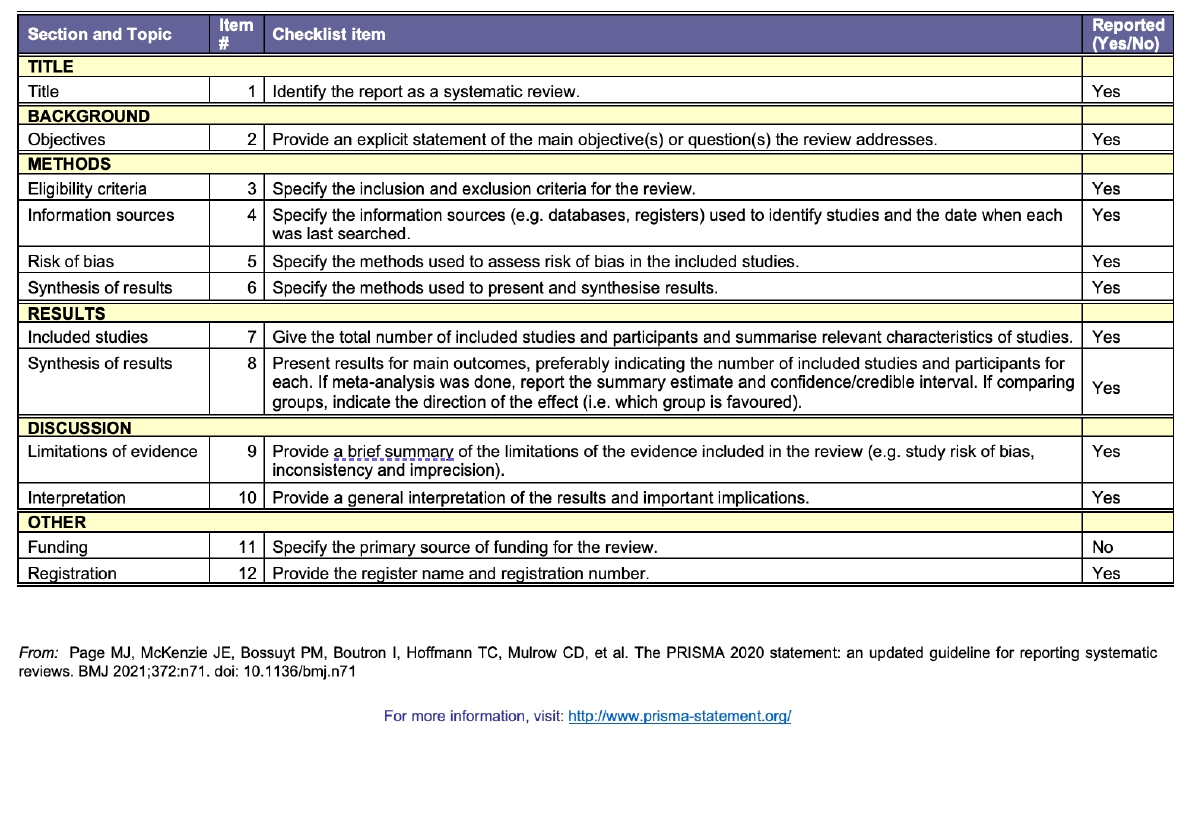

Supplement: Supplementary file 2 [file Image_1.JPEG]

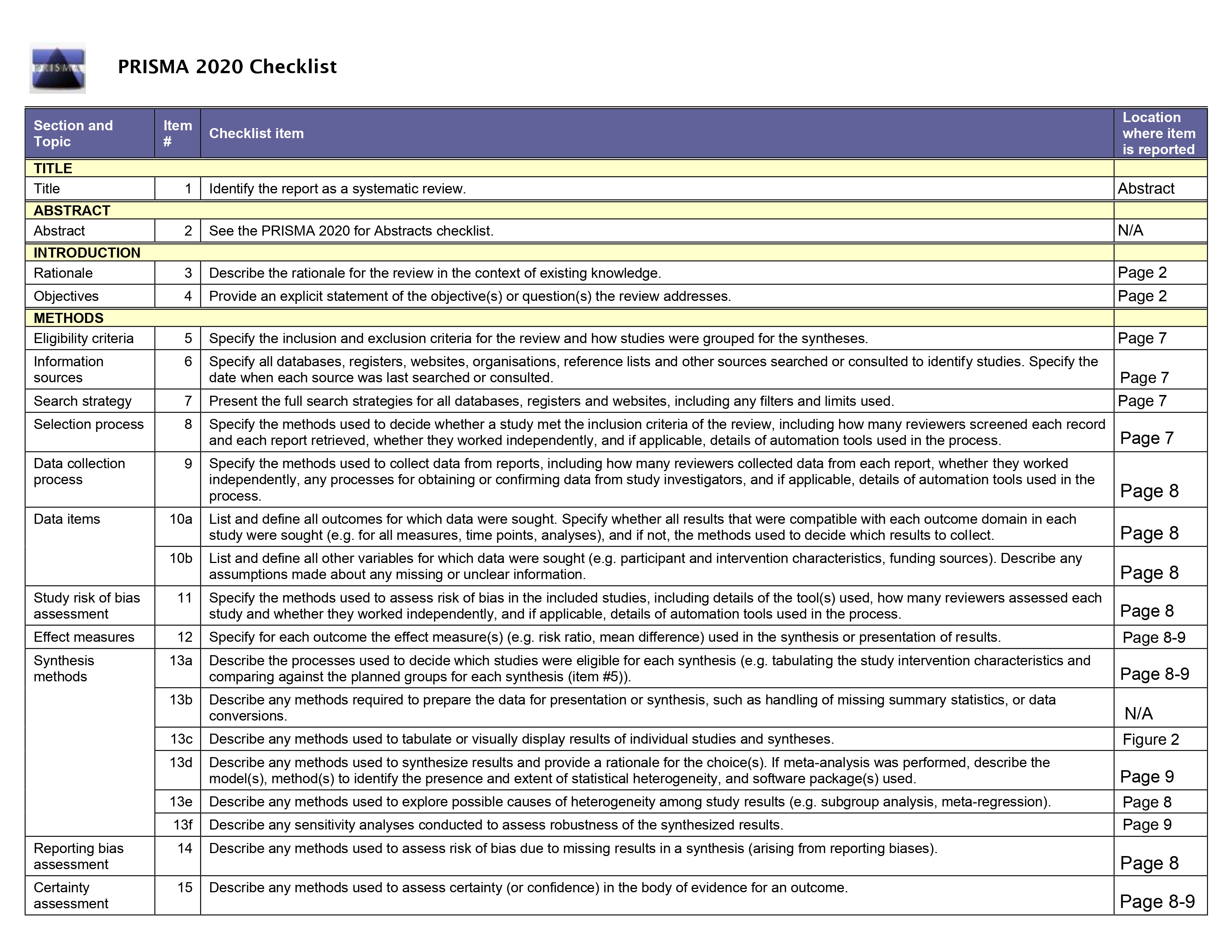

Supplement: Supplementary file 3 [file Image_2.jpeg]

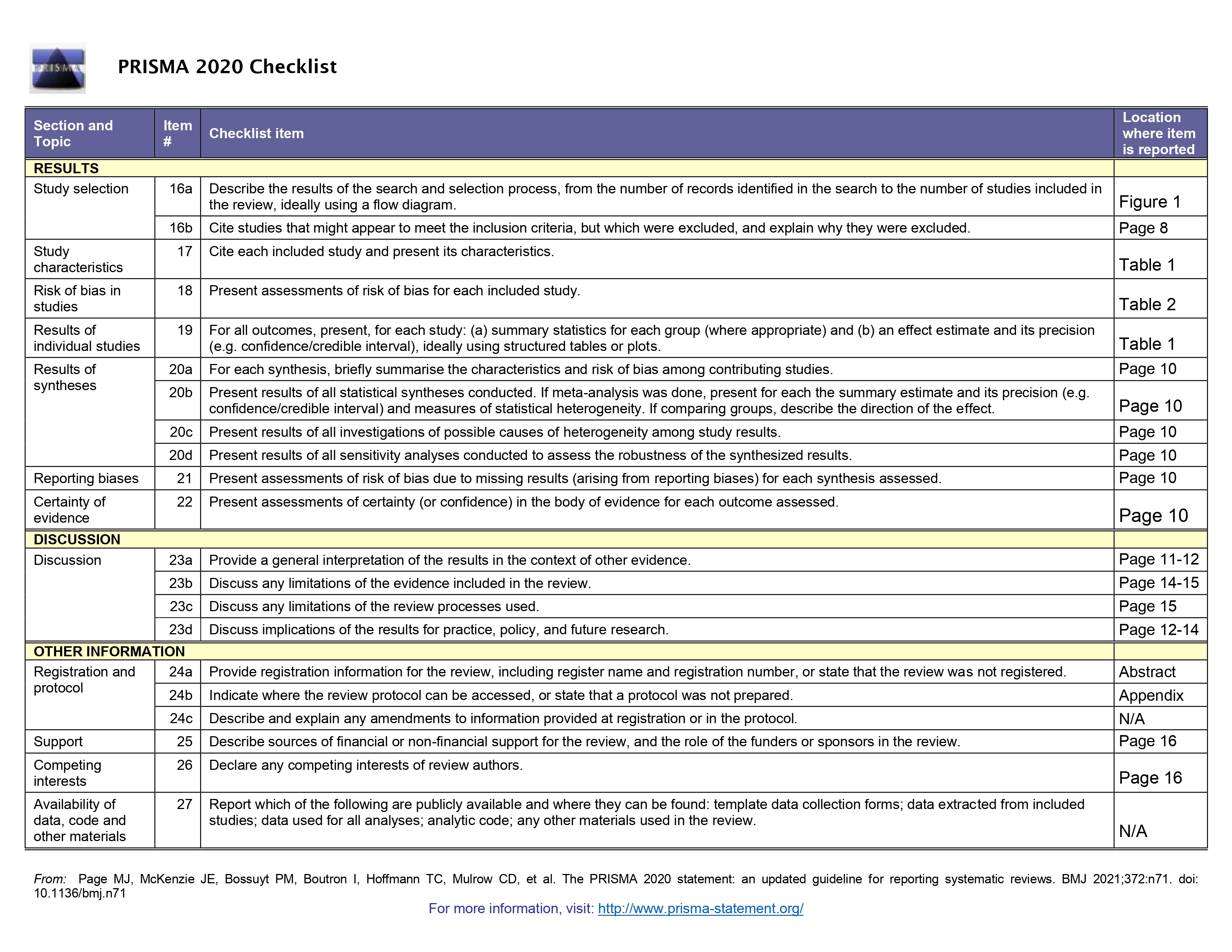

Supplement: Supplementary file 4 [file Image_3.jpeg]

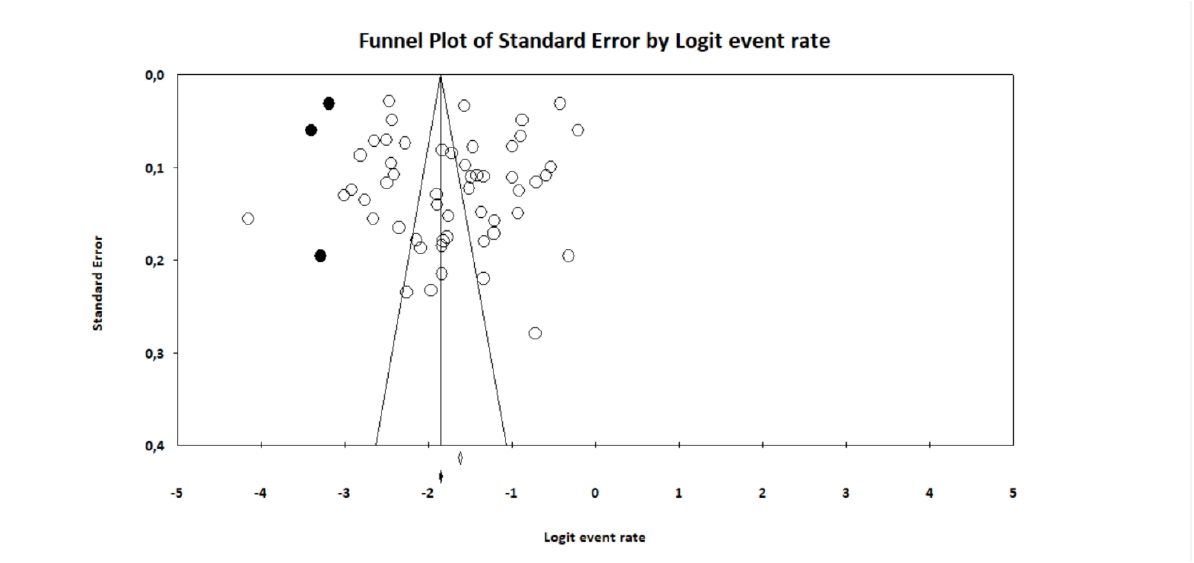

Supplement: Supplementary file 5 [file Image_4.jpeg]
